# Prognosis for Young Females with Pseudomyxoma Peritonei of Appendiceal Origin and Unilateral or Bilateral Ovaries Preserved During Cytoreductive Surgery

**DOI:** 10.3389/fsurg.2022.881510

**Published:** 2022-06-06

**Authors:** Fengxian Fu, Huangdong Tang, Yiyan Lu, Dongmei Lu, Ruiqing Ma

**Affiliations:** ^1^ Department of Gynecology, Aerospace Center Hospital, Beijing, China; ^2^ Department of Myxoma, Aerospace Center Hospital, Beijing, China; ^3^ Department of Pathology, Aerospace Center Hospital, Beijing, China; ^4^ Department of Health Management, Aerospace Center Hospital, Beijing, China

**Keywords:** pseudomyxoma peritonei of appendiceal origin, ovarian involvement, CRS and HIPEC, prognostic prediction, female

## Abstract

**Objective:**

To determine prognosis for young female patients with peritoneal pseudomyxoma (PMP) of appendiceal origin and unilateral or bilateral ovaries preserved during cytoreductive surgery (CRS).

**Methods:**

Clinical data of female patients treated with CRS with or without hyperthermic intraperitoneal chemotherapy (HIPEC) at the Aerospace Center Hospital, Beijing between January, 2009 and December, 2019 were retrospectively reviewed. Patients had no changes in the bilateral ovaries on gross pathological observations or biopsy during CRS, and normal ovarian function. The demographic and clinical characteristics and prognosis of women with ovaries preserved (ovarian preservation group) or resected (ovarian resection group) during CRS were compared. Independent prognostic factors for survival were identified using univariate and multivariate analysis.

**Results:**

40 patients were included in the final analysis. 19 patients chose ovarian preservation while 21 patients underwent ovarian resection. Completeness of cytoreduction (CCR) scores were CCR-0/1. There were significant differences in age (<40 vs. ≥40), symptoms, intraoperative HIPEC (Y vs. N), and histopathologic subtype of PMP (low-grade vs. high-grade) (*p* < 0.001) between patients in the ovarian preservation and ovarian resection groups. In the ovarian preservation group, median overall survival (OS) was 59 months (range, 53–65 months), and the 5-year survival rate was 37.9%. Median disease-free survival (DFS) was 13 months (range, 9–17 months), and the 5-year recurrence rate was 87.4%. In the ovarian resection group, the 5-year survival rate was 87.7%, and the 5-year recurrence rate was 18.3%. Median OS and median DFS were not reached. In patients with low-grade PMP, median DFS was significantly longer in patients with ovarian resection compared to ovarian preservation (*p* < 0.001). Univariate analysis showed histopathologic subtype of PMP (low-grade vs. high-grade, *p* < 0.001) was significantly associated with OS and DFS. On multivariate analysis, high-grade histopathologic subtype of PMP was an independent predictor of poor prognosis (OS and DFS).

**Conclusion:**

Histopathologic subtype of PMP represents an independent predictor of prognosis in female patients with PMP of appendiceal origin and unilateral or bilateral ovaries preserved during CRS. These findings imply that ovarian preservation is a more suitable option for young females with low-grade PMP compared to high-grade PMP. Further prospective studies should be done investigating the role of resection of uninvolved ovaries in PMP.

## Introduction

Pseudomyxoma peritonei (PMP) is a rare clinical syndrome that occurs with an incidence of 2 cases per 100 million individuals (1, 2). Most PMP arise from perforation of a primary appendiceal cancer and seeding of tumor cells within the peritoneal cavity (3). The gold standard curative treatment for PMP is complete cytoreductive surgery plus hyperthermic intraperitoneal chemotherapy (CCRS/HIPEC) (4, 5).

The majority of women with PMP have involvement of the ovaries due to direct invasion from the adjacent appendix or redistribution of PMP within the peritoneal cavity (6, 7); therefore, ovariectomy is often recommended. However, surgical menopause occurs after bilateral ovariectomy. This can have a negative impact on patient quality of life, especially in young women who wish to have children. There remains an unmet clinical need for effective strategies that preserve fertility in young women with PMP of appendiceal origin and to build consensus on management in cases where the ovaries appear normal during CRS. The objective of this study was to determine prognosis for female patients with PMP of appendiceal origin and unilateral or bilateral ovaries preserved during complete cytoreductive surgery (CCRS). Findings will inform clinicians who manage women with PMP.

## Materials and Methods

### Ethical Approval

The protocol for this study was approved by the Ethics committee of the Aerospace Center Hospital, Beijing, China (No. 20161109-ST-07). Written inform consent for publication of clinical data was obtained from all included patients.

### Patient Population

Clinical data of patients with PMP treated at the Aerospace Center Hospital, Beijing between January, 2009 and December, 2019 were retrospectively reviewed. Inclusion criteria were: (1) female; (2) aged 20 to 60 years; (3) diagnosis of PMP of appendiceal origin on histology; (4) initial CRS (radical resection; completeness of cytoreduction [CCR] 0/1) performed at our hospital; and (5) no changes in the bilateral ovaries on gross pathological observations or biopsy during CRS, and normal ovarian function. Exclusion criteria were: (1) PMP derived from other organs or disease (e.g., colon, urachus, and pancreas); (2) previous removal of one or both ovaries; (3) incomplete medical records; or (4) loss to follow-up or death.

**Figure 1:**
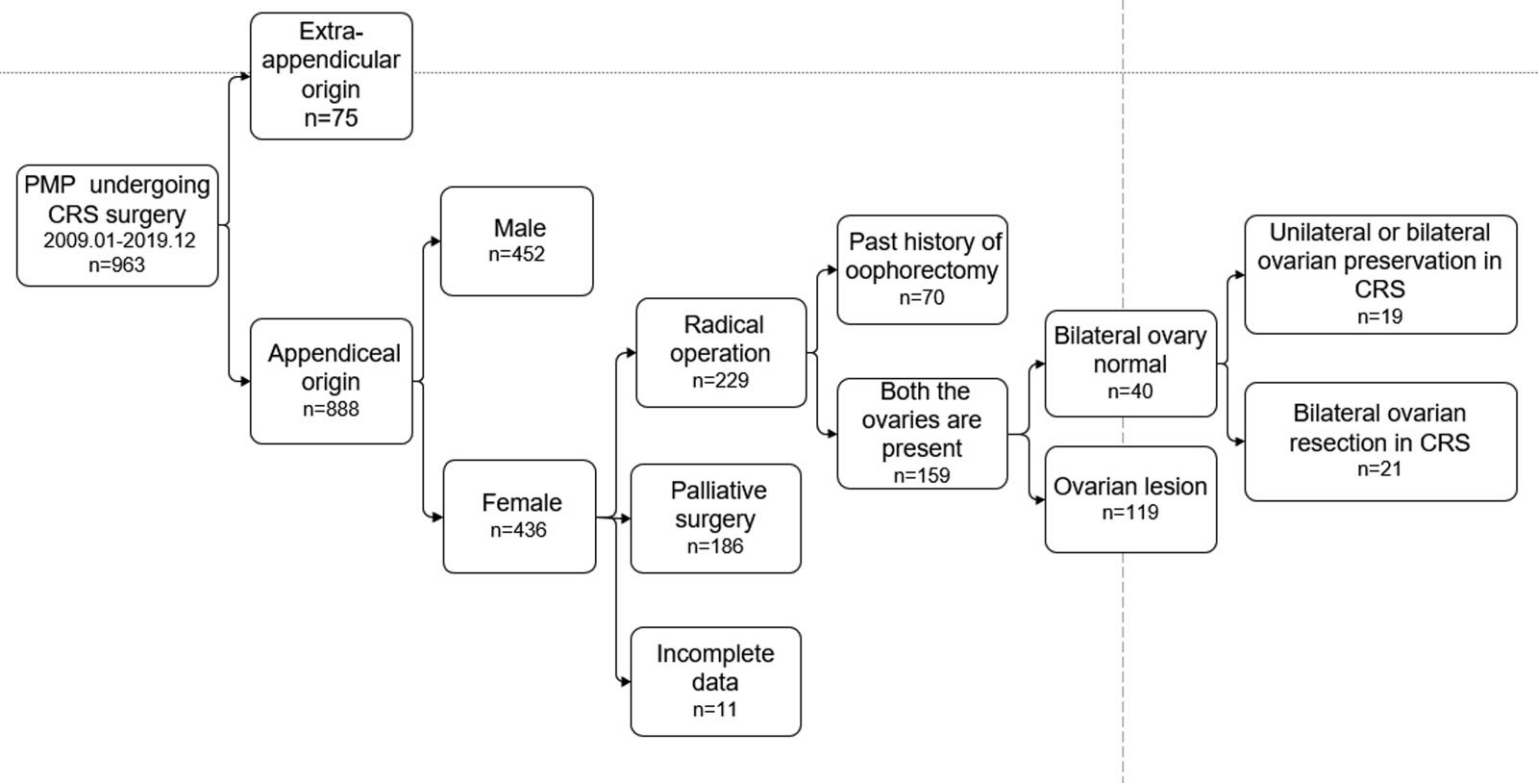
Flow chart of patient selection.

A total of 40 patients were included in the final analysis. Patients were divided into two groups: ovarian preservation group, comprising 19 patients who retained at least one ovary during CRS, and ovarian resection group, comprising 21 patients who underwent bilateral ovariectomy during CRS (Figure 1**)**.

### Surgical Treatment

Patients were treated with CRS with or without HIPEC. Patients underwent CRS to remove visible tumors (6). At our centre, criteria for considering patients for CRS include: (1) aged 20 to 75 years; (2) diagnosis of PMP with histopathologic subtype confirmed by two experienced pathologists; (3) normal liver and kidney function; (3) Eastern Cooperative Oncology Group (ECOG) performance status ≤1; (4) history of severe disease affecting other organs; and (5) presence of distant metastasis or another malignant tumor prior to CRS for PMP.

Patient’s care goals, personal values, and wishes were incorporated into HIPEC decision-making. For patients undergoing HIPEC, inflow and two outflow catheters were placed in the peritoneal cavity and connected to the HIPEC machine (Jilin Minda Company products, China, Model: RHL-2000B). Cisplatin 60–80 mg or mitomycin (20 mg/m^2^) was warmed to 41°C–42°C and circulated intraperitoneally for 60–90 min using a closed-abdomen technique.

### Study Parameters

Patients’ clinicopathological parameters were recorded, including gender; ECOG performance status; age at diagnosis of PMP; symptoms; time from diagnosis of PMP to CRS for PMP; presence/absence of mucus in the abdominal and/or pelvic cavity; intraoperative peritoneal cancer index (PCI); residual disease following CRS measured as CCR; intraoperative HIPEC; pathological grade of PMP; 5-year survival rate; 5-year recurrence rate; Median overall survival (OS); Median disease-free survival (DFS); and follow-up time.

Intraoperative PCI was determined based on tumor size and extent in nine regions in the abdomen and four regions in the small bowel, which were scored on a scale from 0 to 3 and summed (4).

CCR was scored as CCR-0, no macroscopic residual disease, CCR-1, residual disease <2.5 mm, CCR-2, residual disease 2.5 mm–2.5 cm, and CCR-3, residual disease >2.5 cm (5).

Pathological diagnosis was classified according to the 2016 Peritoneal Surface Oncology Group International (PSOGI) criteria (8) as acellular mucin (AC), low-grade mucinous carcinoma peritonei (LG-MCP), high-grade mucinous carcinoma peritonei (HG-MCP), or high-grade mucinous carcinoma peritonei with signet ring cells (HGMC-S).

OS was calculated from the date of CRS/HIPEC to the time of death or last follow-up. DFS was calculated from the date of CRS/ HIPEC to the time of recurrence or last follow-up.

### Statistical Analysis

Statistical analyses were performed using SPSS 24.0 (IBM Corporation, Armonk, NY, USA). Continuous data are expressed as medians and range (min, max). Categorical data are expressed as number and percentages. For categorical variables, data were compared using the χ^2^ or Fisher’s exact test. For continuous variables, normally distributed data were compared with the independent-sample t-test, and non-normally distributed data were compared the Mann-Whitney U test. Independent prognostic factors for survival were identified using univariate survival analysis, which was performed with the Kaplan-Meier method and the log-rank test, and multivariate analysis, which included statistically significant variables in a Cox proportional hazards model. All live patients were censored. *P* < 0.05 was considered statistically significant.

## Results

### Clinicopathological Characteristics

Among 963 patients with PMP who were treated at the Aerospace Center Hospital, Beijing between January, 2009 and December, 2019, 888 (92%) patients had PMP of appendiceal origin, including 436 (49%) female patients and 452 (52%) males. Among the female patients, 229 (52.5%) patients received a radical resection while 186 (42.7%) patients received palliative debulking surgery.

Patients who received a radical resection were eligible for this study. Of these, 70 patients with a history of oophprectomy and 119 (74.8%) patients with ovarian lesions identified during CRS, including 20 patients who had unilateral ovaries preserved, with macroscopic involvement of the other one, were excluded. Finally, 40 (25.2%) patients with bilateral normal ovaries identified during CRS were included in the analysis, including 19 patients who had unilateral (*n* = 4) or bilateral ovarian (*n* = 15) preservation (ovarian preservation group) during CRS and 21 patients who underwent bilateral ovarian resection (ovarian resection group) during CRS (Figure 1).

Patients’ demographic and clinical characteristics are shown in Table 1. In the ovarian preservation group, patients’ median age was 37 years (range, 21–45 years). Median time from diagnosis of PMP to CRS was 1 month. PCI was <20 in 12 (63.2%) patients. Ovarian preservation was bilateral in 15 (78.9%) patients and the left ovary was preserved in 4 (21.1%) patients. CCR scores were CCR-0 in all patients. Pathological diagnosis showed low-grade disease in 10 (52.6%) patients and high-grade disease in 9 (47.4%) patients. In the ovarian resection group, patients’ median age was 53 years (range, 46–59 years). Median time from diagnosis of PMP to CRS was 1 month. PCI was <20 in 19 (90.5%) patients. CCR scores were CCR-0 in all patients. Pathological diagnosis showed low-grade disease in 19 (90.5%) patients. There were significant differences in age (<40 vs. ≥40), symptoms, intraoperative HIPEC (Y vs. N), and histopathologic subtype of PMP (low-grade vs. high-grade) (*p* < 0.05) between patients in the ovarian preservation and ovarian resection groups. There were no patients who needed secondary surgery because of serious complications in ovarian preservation group, but one patient underwent a second operation for urinary fistula in the ovarian resection group. No patients died within 90 days after CRS in two groups.

**Table 1:** Patients’ demographic and clinical characteristics (*n* = 40).

Characteristics	No. of Patients		*p* value
	Ovarian preservation group	Ovarian resection group	
Age at diagnosis (years)			
Median (range)	37 (21–45)	53 (46–59)	<0.001*
<40	12 (63.2%)	0	
≥40	7 (36.8%)	21 (100%)	
Time from diagnosis of PMP to CRS (months)			
<1	8 (42.1%)	11 (52.4%)	0.516
≥1	11 (57.9%)	10 (47.6%)	
Symptoms			
Abdominal distension	5 (26.3%)	1 (4.8%)	0.047*
Appendix neoplasm	3 (15.8%)	3 (14.3%)	
Appendicitis	5 (26.3%)	3 (14.3%)	
Pelvic mass	3 (15.8%)	1 (4.8%)	
Seroperitoneum	1 (.3%)	1 (4.8%)	
Abdominal pain	2 (10.5%)	12 (57.1%)	
Intraoperative HIPEC			
Yes	3 (15.8%)	21 (100%)	<0.001*
No	16 (84.2%)	0	
PCI			
<20	12 (63.2%)	19 (90.5%)	0.369
≥20	7 (36.8%)	2 (9.5%)	
Ovarian Preservation			
Bilateral	15 (78.9%)	0	
Left-side	4 (21.1%)	0	
Right-side	0	0	
CCR post CRS			
0	19 (100%)	21 (100%)	
1	0	0	
Histopathologic subtype			
LG-MCP	10 (52.6%)	19 (90.5%)	0.007*
HG-MCP	9 (47.4%)	2 (9.5%)	
IVCT post CRS			
Yes	3 (15.8%)	4 (19.0%)	0.787
No	16 (84.2%)	17 (81.0)	

*PMP, pseudomyxoma peritonei; CRS, cytoreductive surgery; PSC, previous systemic chemotherapy; PCI, peritoneal cancer index; CCR, completeness of cytoreduction; HIPEC, hyperthermic intraperitoneal chemotherapy; LG-MCP, low-grade mucinous carcinoma peritonei; HG-MCP, high-grade mucinous carcinoma peritonei; IVCT, Intravenous chemotherapy.*

### Fertility Data

In the ovarian preservation group, most ovaries were preserved to maintain hormone production. 6 women were of child bearing age and wished to have children, and one patient was planning to undergo in vitro fertilization. At the end of follow-up, no patient achieved successful childbirth.

### Survival Data

**Figure 2:**
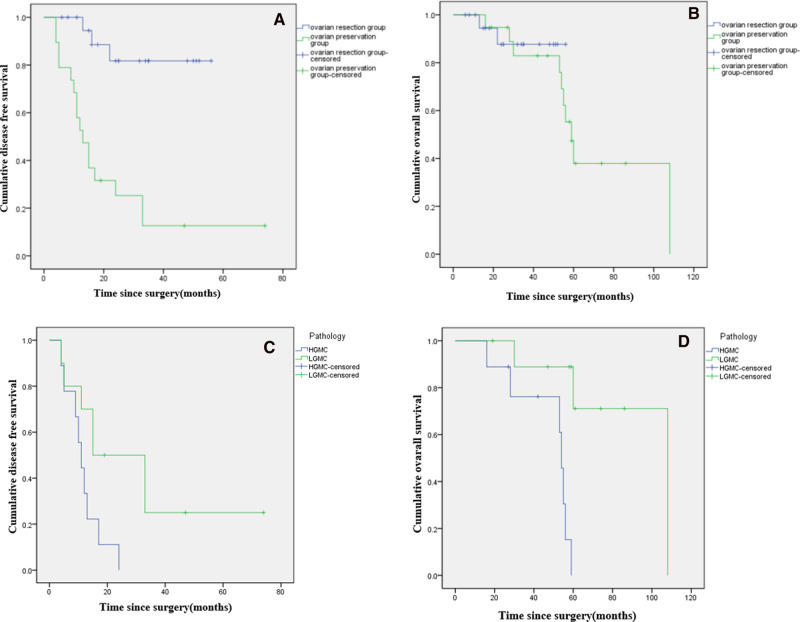
Kaplan-Meier curves showing disease free survival (**A**) and overall survival (**B**) of female patients with PMP of appendiceal origin and unilateral or bilateral ovaries preserved during CRS. Comparison of disease-free survival (**C**) and overall survival (D*) based on histopathologic subtype of PMP (low-grade vs. high-grade). (*: *p *< 0.05).

At the last follow-up in June 2021. In the ovarian preservation group, mean follow-up time was 63 months, 9 (47.4%) patients were alive. 10 patients experienced disease progression. Median OS was 59 months (range, 53–65 months), and the 5-year survival rate was 37.9%. Median DFS was 13 months (range, 9–17 months), and the 5-year recurrence rate was 87.4%. In the ovarian resection group, mean follow-up time was 31 months, 19 (90.5%) patients were alive. 2 patients experienced disease progression. The 5-year survival rate was 87.7%, and the 5-year recurrence rate was 18.3%. Median OS and median DFS were not reached (Figures 2A,B).

In the ovarian preservation group, patients were stratified by histopathologic subtype of PMP (LG-MCP, *n* = 10; HG-MCP, *n* = 9). Among patients with LG-MCP, median OS was 108 months, and the 5-year survival rate was 71.1%. Median DFS was 15 months (range, 0–30 months), and the 5-year recurrence rate was 75.0%. Among patients with HG-MCP, median OS was 54 months (range, 52–56 months), and the 5-year survival rate was 0%. Median DFS was 11 months (range, 8–14 months), and the 5-year recurrence rate was 100.0% (Figures 2C,D).

**Figure 3:**
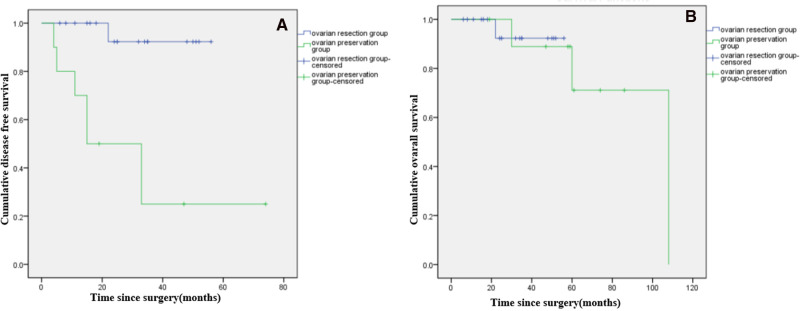
Kaplan-Meier curves showing disease free survival (A*) and overall survival (**B**) of female patients with low-grade PMP of appendiceal origin and unilateral or bilateral ovaries preserved during CRS. (*: *p *< 0.05).

**Figure 4:**
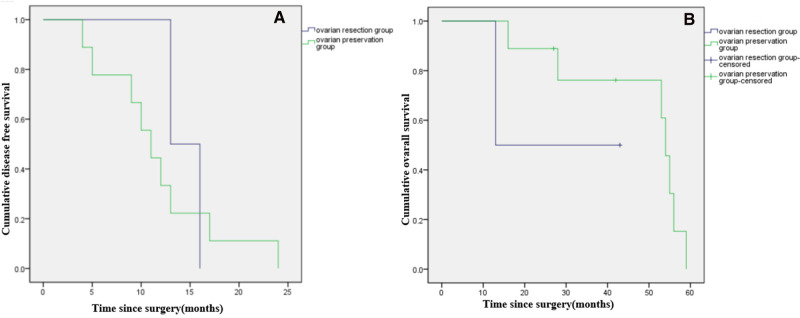
Kaplan-Meier curves showing disease free survival (**A**) and overall survival (**B**) of female patients with high-grade PMP of appendiceal origin and unilateral or bilateral ovaries preserved during CRS.

Among patients with LG-MCP (*n* = 29), median DFS was significantly longer in patients with ovarian resection compared to ovarian preservation (*p *< 0.001), but there was no significant difference in OS (*p* = 0.897). (Figures 3A,B). Among patients with HG-MCP (*n* = 11), there were no significant differences in DFS (*p* = 0.640) or OS (*p* = 0.315) between patients with ovarian preservation and ovarian resection (Figures 4A,B).

Univariate analysis showed histopathologic subtype of PMP (low-grade vs. high-grade, *p* < 0.05) was significantly associated with OS and DFS (Tables 2, 3). On multivariate analysis, high-grade histopathologic subtype of PMP was an independent predictor of poor prognosis (OS and DFS) **(**Tables 2, 3**)**.

**Table 2:** Univariate and multivariate analysis of factors affecting OS (*n* = 40).

Variable	Ovarian preservation group (*n* = 19)			Ovarian resection group (*n* = 21)		
	Univariate *p* value	Multivariate HR (95% CI)	*p* value	Univariate *p* value	Multivariate HR (95% CI)	*p* value
Time from diagnosis of PMP to CRS (<1 vs. ≥1, months)	0.322			0.691		
Mucus in the abdominal cavity (Yes vs. No)	0.639			0.454		
Mucus in the pelvic cavity (Yes vs. No)	0.813			0.929		
Intraoperative HIPEC (Yes vs. No)	0.428			0.528		
PCI (<20 vs. ≥20)	0.089	4.054	0.606	0.929		
		(2.295–7.161)				
Histopathologic subtype (LG-MCP vs. HG-MCP)	0.012	0.076	0.023*	0.103		
		(0.008–0.701)				
IVCT post CRS (Yes vs. No)	0.403			0.403		

***Abbreviations:** PMP, pseudomyxoma peritonei; CRS, cytoreductive surgery; PSC, previous systemic chemotherapy; PCI, peritoneal cancer index; CCR, completeness of cytoreduction; HIPEC, hyperthermic intraperitoneal chemotherapy; LG-MCP, low-grade mucinous carcinoma peritonei; HG-MCP, high-grade mucinous carcinoma peritonei; IVCT, Intravenous chemotherapy.*

**Table 3:** Univariate and multivariate analysis of factors affecting DFS (*n* = 40).

Variable	Ovarian preservation group (*n* = 19)			Ovarian resection group (*n* = 21)		
	Univariate *p* value	Multivariate HR (95% CI)	*p* value	Univariate *p* value	Multivariate HR (95% CI)	*p* value
Time from diagnosis of PMP to CRS (<1 vs. ≥1, months)	0.778			0.274		
Mucus in the abdominal cavity (Yes vs. No)	0.245			0.381		
Mucus in the pelvic cavity (Yes vs. No)	0.204			0.382		
Intraoperative HIPEC (Yes vs. No)	0.174			0.499		
PCI (<20 vs. ≥20)	0.068	1.921	0.215	0.382		
		(0.684–5.397)				
Histopathologic subtype (LG-MCP vs. HG-MCP)	0.038	0.076	0.044*	0.942		
		(0.098–0.968)				
IVCT post CRS (Yes vs. No)	0.493			0.096		

***Abbreviations:** PMP, pseudomyxoma peritonei; CRS, cytoreductive surgery; PSC, previous systemic chemotherapy; PCI, peritoneal cancer index; CCR, completeness of cytoreduction; HIPEC, hyperthermic intraperitoneal chemotherapy; LG-MCP, low-grade mucinous carcinoma peritonei; HG-MCP, high-grade mucinous carcinoma peritonei; IVCT, Intravenous chemotherapy.*

## Discussion

PMP is a clinical entity characterized by mucinous ascites, peritoneal soft-tissue implants, omental caking, and involvement of the gastrointestinal tract and ovaries. A distinguishing feature of PMP is its redistribution within the peritoneal cavity determined by normal flow of peritoneal fluid and gravity. In the present study, among the 963 patients with PMP who were treated at the Aerospace Center Hospital, Beijing between January, 2009, and December, 2019, 92% of patients had PMP of appendiceal origin. This rate is consistent with previously reported case series of patients with PMP treated with CRS/HIPEC, among which 89.6%–94% of patients with PMP had a primary appendiceal tumor (8–10). In other patients, PMP may originate from a tumor in the ovary, colon, small bowel, urachus, pancreas, bile duct, stomach, uterine cervix, fallopian tube, mesentery, kidney, extraovarian teratoma, or spleen.

In females, PMP of appendiceal origin usually metastasizes to the peritoneal surface of the ovaries and the uterus. At our center, the probability of ovarian involvement was 74.8% (119/159). A total hysterectomy with bilateral salpingo-oophorectomy may be recommended, regardless of pre-operative gynecologic organ involvement. However, young women may wish to avoid the associated iatrogenic surgical menopause and permanent infertility (11), which can have major psychosocial consequences and a negative impact on the quality of life of the patient and her family. In the present study, there was a significant difference in age between patients who chose to preserve their ovaries during CRS and those who underwent ovarian resection, with younger patients more likely to choose ovarian preservation.

Among the 19 patients with PMP of appendiceal origin and unilateral or bilateral ovaries preserved during CRS included in our study, 6 desired to have children. At the end of follow-up, only one patient was planning to undergo in vitro fertilization. Our findings showed that PMP recurrence rates rose rapidly two years post-CRS, implying that women who underwent CRS with/without HIPEC for PMP with ovarian preservation should attempt to conceive as soon as possible when the recommended waiting period following therapy is complete (12, 13).

In other literature, two small retrospective studies and 5 case reports have investigated the feasibility of ovarian preservation in patients with PMP of appendiceal origin (14–20). One study in four women aged 28–35 years with PMP who sought to maintain fertility adopted a strategy that involved laparoscopy for disease staging followed by appendicectomy, irrigation of the abdominal and pelvic cavity with water, and stripping of macroscopic disease from the peritoneal surface of the pelvis and the surface of the ovaries. All patients had a low-grade appendiceal mucinous neoplasm with acellular mucin or LG-MCP in the peritoneal cavity. After the procedure, all patients conceived and gave birth to healthy babies. After 12–29 months of follow-up, all women were well with no evidence of disease recurrence on radiology or laparoscopy (21). Other women have conceived following treatment with CCRS + HIPEC. In one study, women aged <41 years with peritoneal carcinomatosis of various origins who expressed a strong desire for future pregnancy were treated with CCRS + HIPEC. At least one ovary was preserved in 21 women. Of these, 4 women developed ovarian recurrence after a median follow-up of 32 months, and two women became pregnant (14). An international survey reported seven pregnancies in women with PMP, epithelial mesothelioma, or papillary mesothelioma who underwent genital organs-preserving CRS and HIPEC, with delivery of seven newborns. Bilateral ovaries were preserved in 5 women, the left ovary was preserved in one woman, and ovocytes were harvested and cryopreserved in one woman. All women were disease free at 42–106 months of follow-up (15). In a case study, a 28-year-old patient with PMP underwent CRS + HIPEC. Bilateral ovaries were preserved, and the woman spontaneously conceived 14 months after surgery. The pregnancy was uneventful (16).

In our study, among all patients, median OS was 71 months (range, 54–88 months), and the 5-year survival rate was 37.9%. In a previous retrospective study of 2,289 patients from 16 specialized units who underwent CRS for PMP, 10- and 15-year survival rates were 63% and 59%, respectively, treatment-related mortality rate was 2%, and major operative complications occurred in 24% of patients (22). In another study of 42 patients who underwent CRS + HIPEC, 5-year survival rates after first and second CRS were 75.5% and 67.7%, respectively (23). In the present study, the incidence of serious complications and mortality rate was acceptable, but 5-year survival rate was comparatively low, potentially due to the distribution of pathological types. In the ovarian preservation group, 47.4% of patients had HG-MCP, which is associated with a poor prognosis. The ovary is a reproductive and endocrine organ that has a rich blood supply, which may promote tumor growth and metastasis. Consequently, we recommend ovarian resection during CRS in patients with HG-MCP. Meanwhile, the limitations section of our study provides context around our patient population, stating that the majority of patients were transferred to our institution from a local hospital in poor general condition, which increased their risk of mortality. Patients in this study were treated with CRS with or without HIPEC, and patient’s care goals, personal values, and wishes were incorporated into HIPEC decision-making. We believe that this treatment pathway is representative of the clinical situation in the real world.

In our study, among all patients, median DFS was 22 months (range, 12–32 months), and the 5- year recurrence rate was 87.4%, which are higher than reported elsewhere (24). Disparate findings between the present study and previous findings may be explained by differences in the patient populations. We included patients with LG-MCP or HG-MCP, while the previous report focused on patients with LG-MCP. Median DFS among our patients with LG-MCP was 32 months (range, 15–49 months), and the 5-year recurrence rate was 75.0%, which were comparable to the previous report. The present study was conducted at a referral center for myxoma, which may have led to selection bias favoring patients with more severe disease. Most notably, PMP is a rare disease; therefore, small sample size may have affected our findings.

In our study, there was no significant difference in OS in patients with LG-MCP or HG-MCP, whether ovariectomy was performed or not; however, in patients with LG-MCP, median DFS was significantly longer in patients with ovarian resection compared to ovarian preservation This suggests that ovarian preservation may increase risk for disease progression, but has little effect on the final prognosis of the patient. On multivariate analysis, high-grade histopathologic subtype of PMP was an independent predictor of poor prognosis (OS and DFS). This may be related to the growth pattern of the tumor cells and ovarian retention. Ovarian involvement is correlated with the peritoneal extent of PMP and tumor grade. Previous studies showed higher rates of ovarian invasion in patients with grade 2–3 PMP (25); specifically, 62% of ovaries were invaded in patients with grade-1 PMP, and 87.5% of ovaries were invaded in patients with grade 2–3 PMP (26). Other studies confirm these findings (27–30). Interestingly, in our study, neither PCI nor the use of HIPEC were independent predictors of prognosis. This may indicate that radical resection is more important than tumor burden and HIPEC in influencing prognosis.

This study was associated with several limitations. First, this was a retrospective study, and several clinical and histopathological data were lacking, such as the histology of the resected ovaries and intravenous chemotherapy regimen. The two groups were not homogenous to some extent. Second, most patients were transferred to our institution from a local hospital in poor general condition, which increased their risk of mortality. Last, the follow-up time was not long enough, especially for patients with preserved ovaries. Further large-scale studies are needed to confirm our results.

In conclusion, histopathologic subtype of PMP represents an independent predictor of prognosis in female patients with PMP of appendiceal origin and unilateral or bilateral ovaries preserved during CRS. These findings imply that ovarian preservation is a more suitable option for young females with low-grade PMP compared to high-grade PMP. However, the reported data were very limited and further prospective studies should be done investigating the role of resection of uninvolved ovaries in PMP.

## Data Availability

The original contributions presented in the study are included in the article/Supplementary Material, further inquiries can be directed to the corresponding author/s.
